# Intraocular pressure reduction in a pigmentary glaucoma model by Goniotome Ab interno trabeculectomy

**DOI:** 10.1371/journal.pone.0231360

**Published:** 2020-04-16

**Authors:** Chao Wang, Yalong Dang, Priyal Shah, Hamed Esfandiari, Ying Hong, Ralitsa T. Loewen, Susannah Waxman, Sarah Atta, Xiaobo Xia, Nils A. Loewen

**Affiliations:** 1 Department of Ophthalmology, University of Würzburg, Würzburg, Germany; 2 Department of Ophthalmology, Xiangya Hospital, Central South University, Changsha, Hunan, China; 3 Department of Ophthalmology, University of Pittsburgh Medical Center, Pittsburgh, Pennsylvania, United States of America; 4 Department of Ophthalmology, Peking University Third Hospital, Beijing, China; Edith Wolfson Medical Center, ISRAEL

## Abstract

**Purpose:**

To investigate whether microsurgical excision of trabecular meshwork (TM) in an ex vivo pigmentary glaucoma model can normalize the hypertensive phenotype.

**Methods:**

Eight eyes of a porcine pigmentary glaucoma model underwent 90° of microsurgical TM excision with an aspirating dual-blade (Goniotome (G)). 24 hours later, additional 90° of TM were removed. Anterior segments with sham surgeries served as the control (C). Outflow facility and intraocular pressure (IOP) were analyzed. Histology with hematoxylin and eosin (H&E) was obtained.

**Results:**

After the first 90° TM excision, IOP was significantly lower in G (10.2±2.4 mmHg, n = 7) than C (20.0±2.0mmHg, n = 8, P<0.01). Outflow facility in G (0.38±0.07 μl/min/mmHg) was higher than C (0.16±0.02 μl/min/mmHg, P<0.01). After the second 90° TM excision, IOP in G (6.46±0.81 mmHg, n = 7) was significantly lower than C (20.3±1.7 mmHg, n = 8, P<0.001), while the outflow facility in G (0.50±0.05 μl/min/mmHg, n = 7) was higher than C (0.16±0.01 μl/min/mmHg, n = 8, P<0.001). Compared to the first excision, excision of an additional 90° did not change of IOP (P = 0.20) or outflow facility (P = 0.17) further.

**Conclusions:**

Excision of 90° of TM in a pigmentary glaucoma model using an aspirating dual-blade decreased IOP and increased outflow facility.

## Introduction

Pigment dispersion syndrome (PDS) can lead to pigmentary glaucoma (PG), a form of secondary open-angle glaucoma, which often affects nearsighted individuals in their 30s to 40s [1, 2]. The prevalence of PDS is as high as 2.5% in the general population and incurs a risk of 15% of PG within 15 years [3]. In PDS, the iris releases cellular debris that contains pigment granules which accumulate in the trabecular meshwork (TM), on the corneal endothelium in the form of the Krukenberg spindle, on the lens surface, and elsewhere [4]. The pathogenesis of PDS is poorly understood, but several mutations or variants of more than one gene seem to contribute with a susceptibility locus at 7q35–q36 [5]. Cytoskeletal and structural TM changes [6] can make PG more challenging to treat medically or by laser [2]. These challenges include IOP spikes and a reduced success rate after trabeculoplasty in heavily pigmented eyes [7, 8]. After a first trabeculoplasty, the need for a second laser or operating room procedure is twice as high in eyes with pigmentary glaucoma [9]. Mid-peripheral iris transillumination defects can be seen early in the course of the disease, which can progress to more extensive iris atrophy.

Trabecular ablation, for instance, by Trabectome surgery (Neomedix Corp., Tustin, California, United States), can be effective in a range of glaucoma disease stages [10–15] which makes it well suited for PG. Ab interno angle surgeries rely on maintaining the anterior chamber either passively with a viscoelastic device [16, 17] or actively, using an irrigation and aspiration (I&A) system [18, 19]. The advantages of a clear angle view and anterior chamber stability have recently become more readily available with an I&A-equipped dual-blade (Goniotome, Neomedix Corp., Tustin, California, United States). This device does not require a high-frequency generator to molecularize the TM and instead excises a strip of TM tissue [16, 20]. These features and its ability to excise TM in a controlled environment and to harvest it non-destructively make it also useful for glaucoma research.

In the present study, we hypothesized that trabecular excision with an I&A-equipped dual-blade device could also restore outflow by removing the pathology. We have extensive experience studying outflow in experimental systems [21, 22], including gene transfer [23–29], disease modeling [6, 30, 31] and surgical outflow enhancement [16, 17, 20, 32–35], but this is the first study of a microsurgical intervention for glaucoma in an ex vivo model of glaucoma.

## Materials and methods

### Pig eye perfusion culture and pigmentary dispersion glaucoma model

Sixteen porcine eyes were obtained from a local abattoir (Thoma Meat Market, Saxonburg, PA) as left-right matched pairs and were processed within two hours of sacrifice. Our Institutional Animal Care and Use Committee (IACUC) assessed that an IACUC protocol, approval or waiver was not required because no animals were sacrificed for the purpose of doing research. After the removal of extraocular tissues, the eyes were decontaminated in 5% povidone-iodine solution (3955–16, Ricca Chemical Company, Arlington, TX 786012) for two minutes and irrigated three times with phosphate-buffered saline (PBS). The posterior segment, lens, iris, and ciliary body were carefully removed. Anterior segments with intact TM were mounted in perfusion dishes as described before [36]. The perfusion media consisted of Dulbecco's modified Eagle media (DMEM, SH30284, HyClone, GE Healthcare, UK) supplemented with 1% fetal bovine serum (FBS, 10438026, Thermo Fisher Scientific, Waltham, MA) and 1% antibiotic/antimycotic (15240062, Thermo Fisher Scientific, Waltham, MA) at a constant rate of 3 μl/min using a microinfusion pump (PHD 22/2000; Harvard Apparatus, Holliston, MA).

A suspension of pigment granules was generated with freeze-thaw cycles, as described previously [6]. In brief, the irises of 10 decontaminated porcine eyes were isolated, frozen at -80C° for two hours, and thawed at room temperature for 2 hours. This process was performed twice to lyse cells and release pigment granules. The lysate was suspended in 15 mL PBS and aspirated and expelled 20 times to promote further pigment release. After filtering through a 70-μm cell strainer (431751, Corning Incorporated, Durham, NC), the suspension was centrifuged at 3000 rpm for 15 minutes. The supernatant was discarded, and the pigment was resuspended in 15 ml PBS. The centrifugation and resuspension steps were repeated four times. The pigment pellet was resuspended in 4 mL PBS for pigment stock solution. The stock solution was diluted 1000-fold, and concentration was determined with a hemocytometer, using 600x magnification (Eclipse TE200-E, Nikon Instruments Inc., Melville, NY). Pigment granules were added to perfusion media after 48h of IOP stabilization, at a concentration of 1.67×107 particles/mL. The IOP was measured with pressure transducers (SP844; MEMSCAP, Skoppum, Norway) and recorded every two minutes (LabChart, ADInstruments, Colorado Springs, CO).

### Trabecular meshwork excision

**Fig 1:**
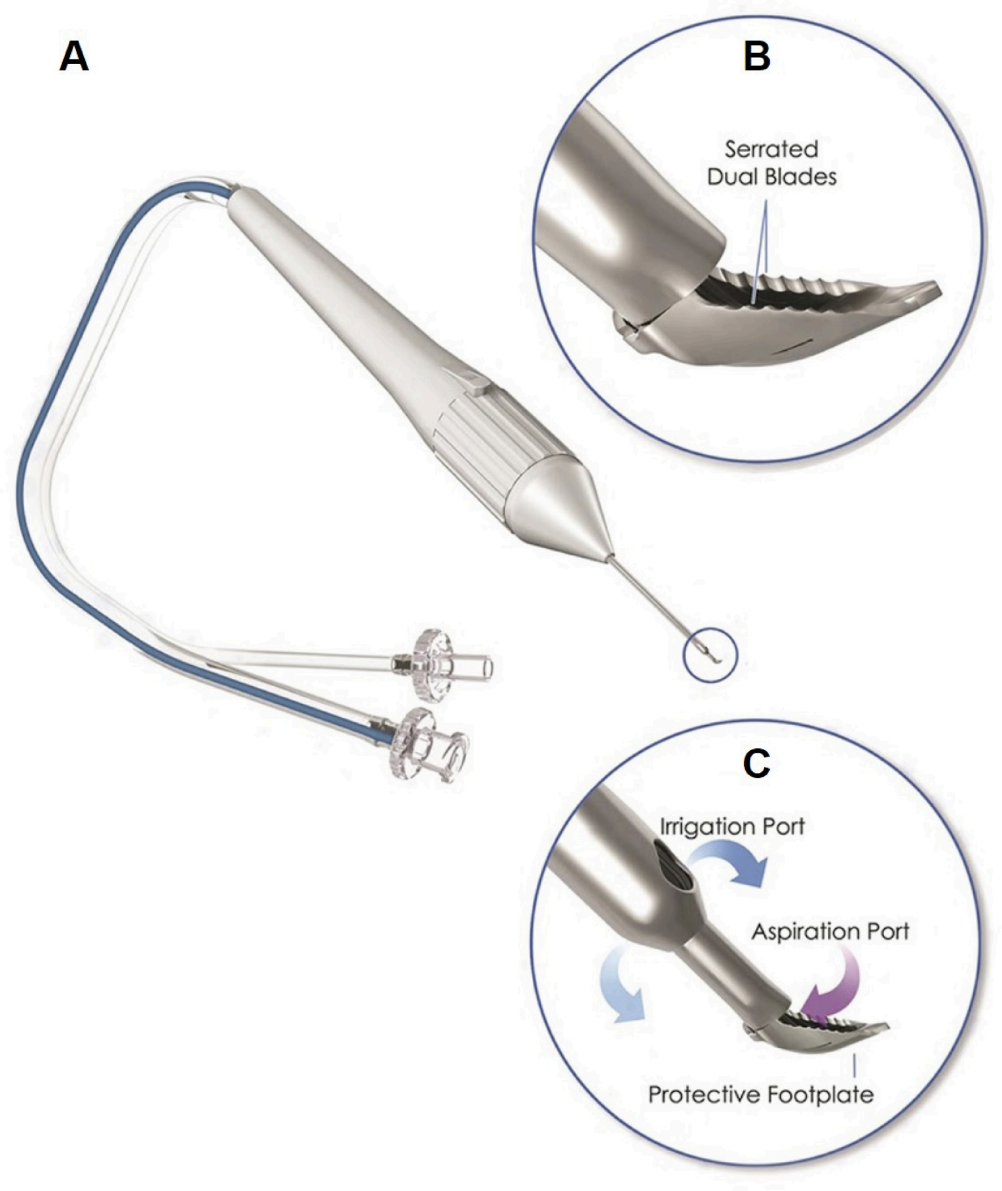
Goniotome surgical system. The Goniotome has two irrigation ports that maintain the chamber (C, blue arrows). The TM is put under stretch by a ramp and excised. TM, blood, and debris are aspirated into the tip (C, red arrow).

When the hypertensive plateau was established, the TM in experimental group G was excised with the Goniotome (Neomedix Corp., Tustin, CA, United States, Fig 1). A single surgeon (NAL) with comprehensive experience in ab interno trabeculectomy performed the procedures. The inverted anterior segments were positioned under a surgical microscope (S4, Carl Zeiss Meditec, Jena, Germany). The tip of the instrument was inserted into the TM, and aspiration was started, causing the serrated dual-blades to engage and excise the TM. The excision was continued 90° to the left, and the excised TM strip was cut by angulating the tip of the Goniotome. After 24 hours, an additional 90° of the TM was removed using the same procedure with an excision path in the opposite direction. Control eyes in group C underwent a sham procedure meant to mimic irrigation and aspiration. Irrigation and aspiration are part of a procedure termed “trabecular aspiration” that has been observed to remove pigmentary debris and induce trabecular meshwork remodeling with a small and transient IOP reduction [37]. As in group G, the Goniotome was placed into the inverted anterior segment under the microscope. Irrigation and aspiration were started, and movements along the TM 90 degrees into each direction were made but without touching the TM.

### Histology

After the perfusion culture experiments, the anterior segments were fixed with 4% paraformaldehyde for 24 hours, rinsed, embedded in paraffin, and sectioned at a thickness of 6 microns. Hematoxylin and eosin staining was performed for gross histological evaluation.

### Statistics

Data were reported as the mean ± standard error unless stated otherwise. A one-way ANOVA was used to compare the data for different groups at individual time points. Paired t-tests were used for an in-group comparison of IOP and outflow facility before and after treatment (PASW 18.0, SPSS, Armonk, New York, United States). Differences were considered statistically significant for P<0.05. IOPs were averaged over 6 hours following excision. Media and dish handling were scheduled to occur outside of these periods to avoid artifacts. The outflow facility was computed using the Goldmann equation, assuming that the episcleral venous pressure is near zero, Po = (F/C) + Pv (Po: IOP, F: rate of aqueous formation, C: outflow facility, Pv: episcleral venous pressure).

The minimum number of eyes for an adequate testing power was determined with a sample size calculator. A minimum sample size of three was required to detect at least an 18% IOP reduction [38] with an alpha of 0.05 and a power of 0.80, given the historical baseline facility of 0.25+/- 0.06 [6]. Our perfusion system allows us to run eight eyes in parallel, and this number was chosen for sufficient redundancy.

## Results

**Fig 2:**
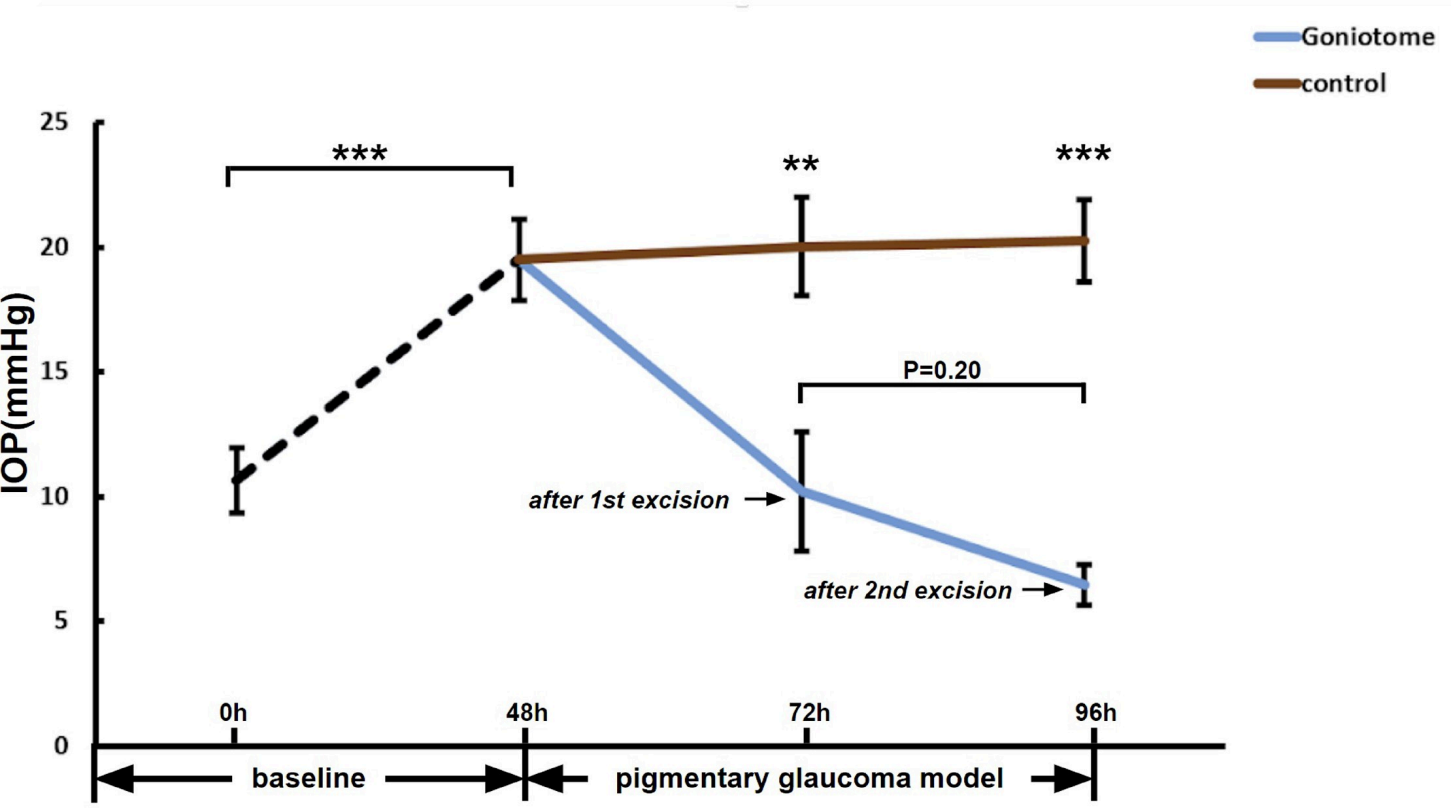
IOP reduction after TM excision. After 48h perfusion with pigment, IOP in PG was significantly higher compared to baseline (n = 15, ***P<0.001). Twenty-four hours after a 90° excision of TM (G), IOP in G (n = 7) was lower than in C (n = 8, **P<0.01). Twenty-four hours after a second, adjacent excision of 90° TM, IOP in G appeared to slightly reduce further (n = 7, P>0.05) remaining lower than C (n = 8, ***P<0.001).

Sixteen porcine eyes were used (eight eyes in C, and eight eyes in G). One eye in G was excluded due to a transducer error. After 48h of perfusion, a stable baseline IOP of 10.7±1.3 mmHg (all eyes) was achieved without a significant difference in IOP between C (11.4±0.6) mmHg and G (9.8±2.7 mmHg; C versus G: P = 0.54). After 48h of pigment perfusion to establish the pigmentary glaucoma model, IOP increased by 82% to a plateau at 19.5±1.6 mmHg (all eyes, P<0.001). At this point, pretreatment IOP in C (21.6±2.6 mmHg) was similar to G (17.7±2.0 mmHg, P = 0.24). The first TM excision in G reduced the IOP to 10.2±2.4 mmHg (n = 7) 24 hours later compared to 20.0±2.0 mmHg in C (n = 8, P<0.01). The second TM excision, now had an average in G of only 6.5±0.8 mmHg, (n = 7, P = 0.20) compared to 20.3±1.7 mmHg (n = 8, P<0.001) in C (Fig 2). The first excision resulted in an IOP reduction of 48% and the second one in an IOP reduction of 67% compared to the hypertensive baseline.

**Fig 3:**
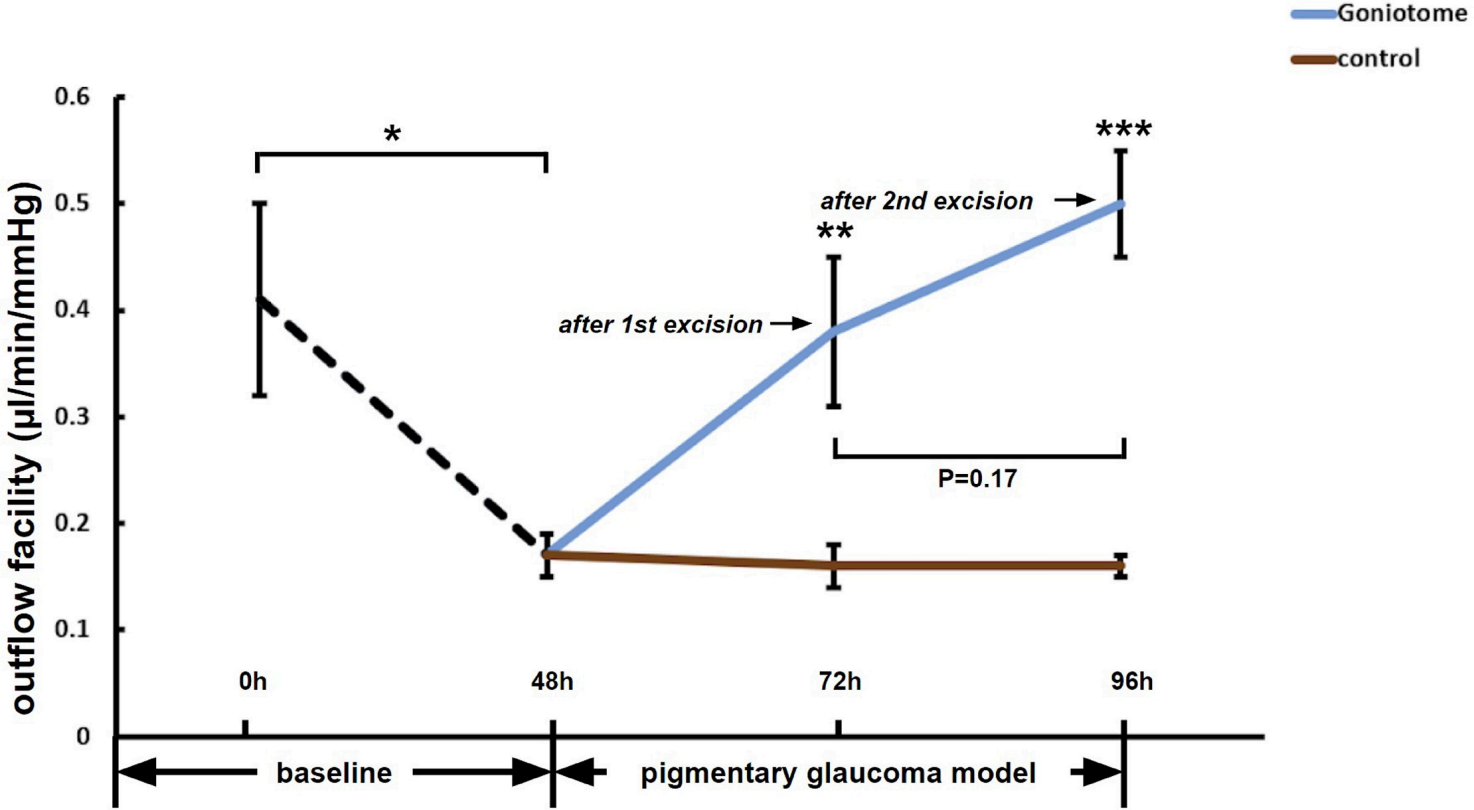
Increased outflow facility after TM excision. The outflow facility of PG was decreased from baseline (n = 15,*P<0.05) after 48 hours of perfusion with pigment. Twenty-four hours after the first 90° TM excision, the outflow facility in G (n = 7) increased compared to C (n = 8, **P<0.01). Twenty-four hours after the second 90° excision, the outflow facility in G (n = 7) remained significantly higher than C (n = 8, ***P<0.001).

After 48h of perfusion, the baseline outflow facility was stable (0.41±0.09, n = 15). After 48h of pigment perfusion, the outflow facility of PG (0.17±0.02, n = 15, P<0.05) was significantly decreased from baseline. 24 hours after the first 90° of TM excision, outflow facility in G (0.38±0.07 μl/min/mmHg, n = 7) was higher than C (0.16±0.02 μl/min/mmHg, n = 8, P<0.01). Twenty-four hours after the second TM excision, outflow facility in G (0.50±0.05 μl/min/mmHg, n = 7) was higher than C (0.16±0.01 μl/min/mmHg, n = 8, P<0.001), but there was no significant further increase in outflow facility after second TM excision (Fig 3), compared with the outflow facility after first TM excision (P = 0.17).

**Fig 4:**
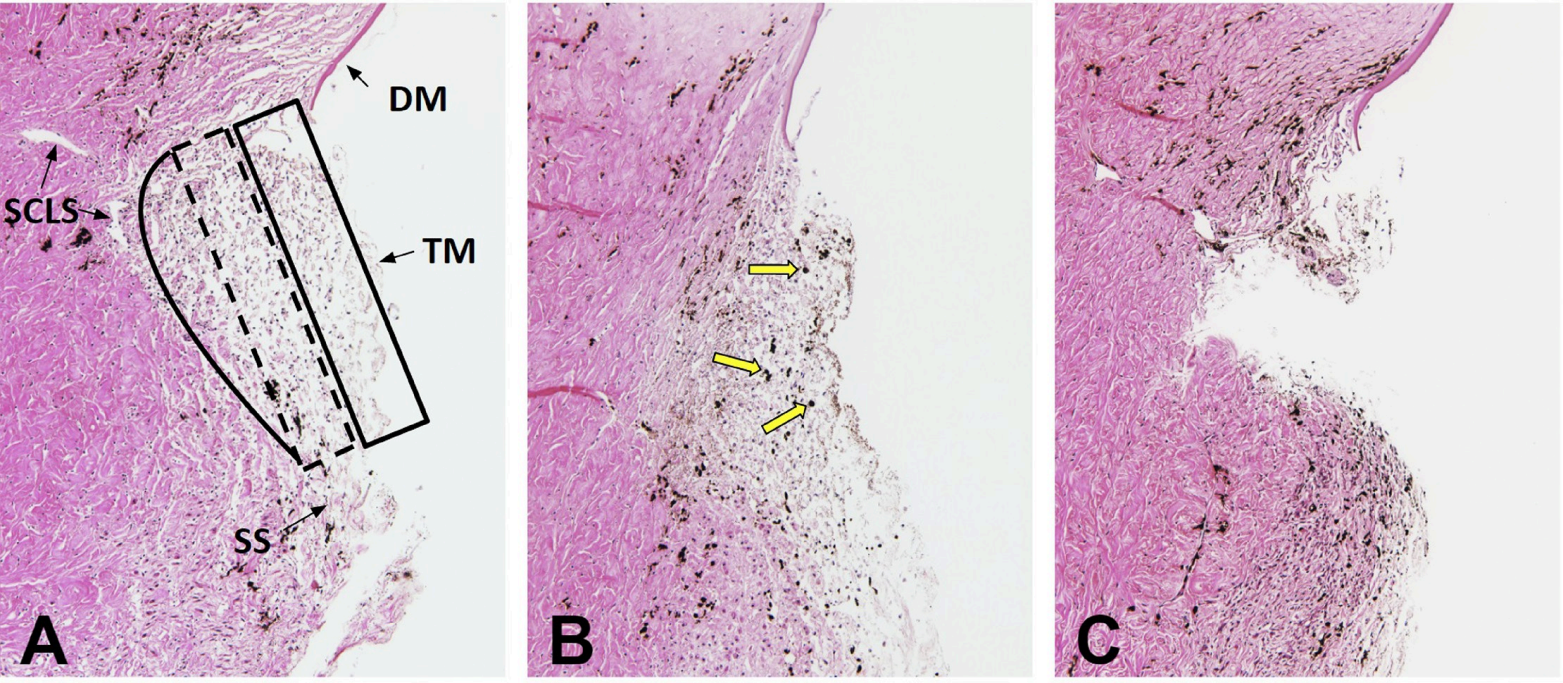
Histology. Normal TM was lightly pigmented and consisted of three areas with different compactness and cell densities: the uveal meshwork (box with solid line, A), the corneoscleral meshwork (box with dashed line, Fig 4A), and the juxtacanalicular meshwork (solid line, A) adjacent to small canal-like segments characteristic of the porcine angular aqueous plexus (SCLS). Pigment granules were observed in all layers of TM (B, yellow arrows) after perfusion with pigment-supplemented media. After TM excision, a large, full-thickness portion of TM was removed (C). Trabecular meshwork: TM, Schlemm’s canal-like segments (SCLS) of the porcine angular aqueous plexus, scleral spur (SS), Descemet's membrane (DM).

The control TM appeared as a thick, multilayered structure consisting of the uveal, the corneoscleral, and the juxtacanalicular meshwork (Fig 4A). Pigs have an angular aqueous plexus without a single lumen Schlemm’s canal characteristic for primate eyes. Instead, smaller, Schlemm’s canal-like segments (SCLS) adjacent to the juxtacanalicular meshwork could be made out. The normal TM was lightly pigmented (Fig 4A). After perfusion with pigment-supplemented media for 48 hours, pigment granules were present in the TM at a density of approximately 10,000-fold below the amount expected to cause a physical obstruction [39]. The pigment granules were seen throughout the TM (Fig 4B, yellow arrows). After the TM excision with the Goniotome, a full-thickness portion of TM appeared removed without damage to the adjacent sclera or corneal endothelium (Fig 4C). In comparison to the smaller human TM that can be removed in its entirety, remnants of adjacent TM remained.

## Discussion

This is the first study of a microincisional intervention in an ex vivo model of glaucoma. We found that trabecular excision with a novel dual-blade device, the Goniotome, induces a 52.5% IOP reduction after only 90° of TM are removed. Additional TM excision did not significantly lower the IOP. This finding is concurrent with the clinical observation in human eyes that the length of TM ablation beyond 90° does not lower IOP more even though it might improve the chances of successfully connecting to working collector channels [40, 41]. Pig eyes do not have a single, contiguous Schlemm’s canal, yet a circumferential flow pattern can be observed here as well [22, 32].

The trabecular meshwork and the inner wall of Schlemm’s canal are primary sites of outflow resistance in primary open angle glaucoma [42], but distal outflow resistance also appears to play a role [43, 44]. In contrast, secondary open angle glaucomas are caused by pathologies mostly limited to the TM and respond well to ab interno trabeculectomy [45–48]. Pigmentary glaucoma is such a secondary glaucoma and tends to present with a relatively high mean IOP of 30 mmHg with a range from 24 to 56 mmHg [3, 49]. The mechanism of outflow compromise in PG is not a simple mechanical obstruction but seems due to a rearrangement and contraction of the TM actin cytoskeleton [30]. The phagocytic overload eventually leads to TM cell death, and collapse and fusion of denuded trabecular beams [50, 51].

Recent evidence points towards a central role of RhoA in regulating the cytoskeleton, motility, and phagocytosis of TM cells in PG [6, 52]. Ocular hypertension emerges as soon as cytoskeletal changes occur, and before phagocytosis declines [30]. We found that a Rho-kinase inhibitor can relax the contracted cytoskeleton and normalize outflow in an ex vivo PG model [31]. In a separate study, we observed that Rho-kinase inhibitors could cause an IOP reduction independent of the TM by dilating the distal outflow vessels. Gottanka et al. described downstream changes in PG that included star-shaped cells migrated away from the TM with an accompanying loss of connecting fibrils [51]. Neither we nor Gottanka et al. nor Alvarado et al. in an earlier study [50] found pigment downstream of the TM. The fact that TM excision in our study increases the facility to a supraphysiological level points again to the TM as the principal pathology.

Non-surgical treatment of PG is challenging [2, 53], and in the past, about 35% of all PG patients needed traditional filtering surgery to control IOP [54]. The safety and efficacy of ab interno trabeculectomy are especially attractive for younger patients, including ones affected by PG. As seen in this model, the continued pigment dispersion does not appear to block exposed collector channel orifices or cause a subsequent IOP rise.

There are several limitations to our study. We used an *ex vivo* model, so immunologic responses, changes of the distal outflow tract distal, and wound healing processes that could affect the IOP, are not taken into account. The observation time as relatively brief. Moreover, there are anatomical differences between human and porcine eyes that may result in a different IOP response.

In conclusion, in this porcine ex vivo PG model, Goniotome surgery caused a 52.5% IOP reduction after removal of 90° of TM.

## Supporting information

S1 File Raw data of experimental groups. (PDF)
