# Supplementary material for: Intraocular pressure reduction in a pigmentary glaucoma model by Goniotome Ab interno trabeculectomy
Source: PLoS One. 2020 Apr 16;15(4):e0231360. doi: 10.1371/journal.pone.0231360 (PMC7162452; doi:10.1371/journal.pone.0231360)
Supplement: S1 File — (PDF) [file pone.0231360.s001.pdf]

	C			
	baseline	48h	72h	96h
	14,2	21,3	28,0	25,0
	10,8	24,6	26,3	24,1
	10,6	24,6	21,0	20,0
	12,6	12,8	14,7	17,7
	11,6	20,5	23,8	27,0
	12,3	14,1	16,2	16,1
	11,3	11,1	13,0	13,5
	8,1	12,5	17,2	18,6
mean	11,4	17,7	20,0	20,2
SE	0,6	2,0	2,0	1,7
	G			
	baseline	48h	72h	96h
	13,6	24,8	7,6	7,1
	5,2	22,4	15,9	7,6
	1,9	8,6	5,6	5,1
	5,6	29,4	22,0	4,6
	18,7	20,5	8,3	5,6
	4,0	18,8	7,6	10,6
	19,3	26,9	4,6	4,8
mean	9,8	21,6	10,2	6,5
SE	2,7	2,6	2,4	0,8
combined baseline				
mean	10,7			
SE	1,3			
